# Globalization, Global Health, and Disaster

**DOI:** 10.3389/fpubh.2015.00262

**Published:** 2015-11-20

**Authors:** Rajendra Karkee

**Affiliations:** ^1^School of Public Health and Community Medicine, BP Koirala Institute of Health Sciences, Dharan, Nepal

**Keywords:** globalization, global health, earthquake, Nepal, disaster

Although Globalization is not a new phenomenon, it has been accelerating in recent years. The world is now more than ever interconnected and interdependent with flows of goods, capital, people, and ideas. This process has had positive as well as negative health effects (1), has become an important health determinant, and has accounted for the emergence of new concepts of “global health” (2). The importance of Globalization and its effect on global health often becomes highlighted during disasters, as in the case of a large scale earthquake or the emergence of a new infectious disease.

If a disaster occurs in low and middle income countries, such countries often do not have the capacity to handle an immediate response in terms of rescue, treatment, and shelter. This can result in inadequate management of affected people and environment and can lead to further disasters including epidemics and social instability, which in turn may affect global health (3).

In this era of Globalization, it is possible to have a timely global response after the occurrence of a disaster and to provide substantial help to affected countries and communities to restore and maintain health services. After a magnitude 7.8 earthquake in Nepal on April 25, 2015, there was just such a response. This disaster caused 8,659 deaths and over 100,000 injuries with more than 500,000 houses and 1,000 health facilities destroyed. The health facilities included primary health care centers, village health posts, and birthing centers (4).

Nepal is in a risk prone area for earthquakes and its capital, Kathmandu, is classified as being very vulnerable should a strong earthquake occur. Kathmandu has seen much unmanaged urbanization, mainly due to centralized resources and failure to enforce even existing laws. Urbanization is another challenge to global public health in the twenty-first century. Cities have been growing unsustainably with poor roads and urban transport, and inadequate water and waste disposal systems. In Kathmandu, many buildings have been constructed without regard to proper engineering principles and people frequently compound the problem by adding floors to houses with weak foundations (5).

A national preparedness platform does exist in Nepal: The Disaster Preparedness Network Nepal consists of governmental and non-governmental organizations. The Network coordinates and shares information to manage disasters such as earthquakes, floods, droughts, landslides. But while a “National Strategy for Disaster Risk Management in Nepal” has been formulated, this strategy does not include separate plans for each type of disaster (6). Because of the likelihood of an earthquake, there has been earthquake preparedness planning by some municipalities with co-ordination and support from various non-governmental organizations. However, this preparedness proved quite inadequate during the recent earthquake experience. This is because planning was limited to minimal training, assessment studies, and paper plans rather than actual storage of sufficient emergency materials and of the rehearsal of a proper co-ordination plan. This is the situation not only in Nepal but also in many other low income countries. Disaster preparedness is made more difficult by a weak central government and inadequate material resources. Thus low income countries need global assistance to plan and prepare for disasters and to manage recovery.

There was a substantial effort by many in the global community to help with Nepal’s earthquake experience. The first rescue team arrived on the day of the earthquake. In the following 2 weeks, 76 Urban Rescue and Search Teams arrived including 2,242 personnel and 135 rescue dogs from 31 countries (7). They rescued hundreds of people, treated thousands more, and distributed food and shelter materials in urban areas. An estimated 330 non-governmental organizations also conducted some 2,200 humanitarian activities during that 2-week period. The activities focused on rescuing trapped people, removing debris, providing shelter, treatment and food items, and keeping the environment tidy.

Of the estimated US$ 422 million required to help the affected people for the initial 5 months, US$ 119.6 million was quickly received from donor countries (4). A Facebook crowd sourcing campaign was able to raise $15 million from 750,000 people around the world within a week (8). All this assistance was possible because of the Globalization of economies, transport, and communication. Without this global help, Nepal could not have coped as well as it did. Many fewer would have been rescued quickly enough to enable their survival. Many of those injured would have died due to lack of treatment and others would have been left to live without shelter or adequate food or water. Thus the effects of the disaster, including deterioration of public health, would have been far worse.

The arrival of global support after a disaster does present management and co-ordination challenges. This was evident in Nepal’s earthquake event as there was confusion about who should command and co-ordinate the relief plan among government institutions, and whether national and international non-governmental organizations should be allowed to work and distribute relief materials independently (9). It is well established that a sector-wide approach to developmental work is most effective and this should be led by the host government. Ideally, all resources for a “sector” should be pooled in a single fund (10). However, disaster and emergency relief is a special situation and needs immediate response and distribution of relief materials. The host government may not be capable of rapid mobilization and its system may be slow in reaching the affected communities. Also, national and international organizations often like to work independently but all work and help should be transparent, communicated to and co-ordinated with governmental institutions as much as possible.

In conclusion, the recent magnitude 7.8 earthquake in Nepal caused massive destruction. An immediate effective response in terms of rescue, treatment, and shelter was beyond the capacity of the government alone. But in this era of Globalization, rapid aid is available to help deal with the disaster and lessen its risk to global health.

## Conflict of Interest Statement

The author declares that the research was conducted in the absence of any commercial or financial relationships that could be construed as a potential conflict of interest.
